# Wastewater-Based Epidemiology (WBE) and Viral Detection in Polluted Surface Water: A Valuable Tool for COVID-19 Surveillance—A Brief Review

**DOI:** 10.3390/ijerph17249251

**Published:** 2020-12-10

**Authors:** Maria de Lourdes Aguiar-Oliveira, Aline Campos, Aline R. Matos, Caroline Rigotto, Adriana Sotero-Martins, Paulo F. P. Teixeira, Marilda M. Siqueira

**Affiliations:** 1 Laboratory of Respiratory Viruses and Measles, IOC, Oswaldo Cruz Foundation-RJ, National Reference Laboratory for Influenza and COVID-19 for the Brazilian Ministry of Health (MoH) and World Health Organization (WHO), Av. Brasil, 4365 Manguinhos, Rio de Janeiro CEP 21040-360, Brazil; aline.matos@ioc.fiocruz.br (A.R.M.); mmsiq@ioc.fiocruz.br (M.M.S.); 2 State Center for Health Surveillance, Rio Grande do Sul State Department of Health. Av. Ipiranga, 5400, Porto Alegre CEP 90610-000, Rio Grande do Sul, Brazil; aline-campos@saude.rs.gov.br; 3 Laboratory of Molecular Microbiology, Feevale University, ERS-239, 2755, Novo Hamburgo CEP 93525-075, Rio Grande do Sul, Brazil; rigotto@feevale.br; 4 Department of Sanitation and Environmental Health, National School of Public Health Sergio Arouca (ENSP), Oswaldo Cruz Foundation-RJ, Av. Brasil, 4365 Manguinhos, Rio de Janeiro CEP 21040-360, Brazil; adrianasotero@ensp.fiocruz.br; 5 Former World Health Organization WHO/PAHO Regional Advisor on Water and Sanitation, Environmental Health, Porto Alegre CEP 90035-002, Rio Grande do Sul, Brazil; pfpiza@yahoo.com

**Keywords:** SARS-CoV-2, wastewater-based epidemiology, wastewater, surface water, fecal–oral transmission, public health

## Abstract

SARS-CoV-2 is the causative agent of the current COVID-19 pandemic. Disease clinical manifestations range from asymptomatic to severe multiple organ damage. SARS-CoV-2 uses ACE2 as a cellular receptor, which is abundantly expressed in the small intestine, allowing viral replication in the gastrointestinal tract. Viral RNA has been detected in the stool of COVID-19 patients and viable viruses had been isolated in some of these samples. Thus, a putative role of SARS-CoV-2 fecal-oral transmission has been argued. SARS-CoV-2 is shed in human excreta and further disposed in the sewerage or in the environment, in poor basic sanitation settings. Wastewater-based epidemiology (WBE) is a valuable population level approach for monitoring viral pathogens and has been successfully used in different contexts. This review summarizes the current global experience on SARS-CoV-2 WBE in distinct continents and viral detection in polluted surface water. The advantages and concerns of this strategy for SARS-CoV-2 surveillance are discussed. Outcomes suggest that WBE is a valuable early warning alert and a helpful complementary surveillance tool to subside public health response, to tailor containment and mitigation measures and to determine target populations for testing. In poor sanitation settings, contaminated rivers could be alternatively used as a source for environmental surveillance.

## 1. Introduction

The severe acute respiratory syndrome coronavirus 2 (SARS-COV-2) is the causative agent of the coronavirus disease 2019 (COVID-19), responsible for one of the major pandemics from the last centuries. After the identification of the first Wuhan cases in December 2019 [1,2], viral dissemination through Asian and European countries led the World Health Organization (WHO) to declare the COVID-19 pandemic in March, 2020 [3]. Currently, the epidemiological scenario varies among countries, according to their epidemic phase and mitigation measures [4].

Clinical manifestations of SARS-CoV-2 infections range from asymptomatic or mild disease to severe pneumonia, with further multiple organ damage [5,6]. Part of COVID-19 patients report gastrointestinal (GI) symptoms, such as diarrhea, nausea, abdominal pain and vomiting [7,8], even in the absence of respiratory symptoms [9]. As SARS-CoV-2 uses angiotensin-converting enzyme 2 (ACE2) as its cellular receptor [10], which is abundantly expressed in the small intestine [11], GI tract is permissive to viral replication. Indeed, the virus was already detected in gastric, esophagus, stomach, duodenum, rectal and intestinal epithelia [12,13,14]. Additionally, viral RNA was detected in the stool or anal swabs of 10.1–82.0% of infected patients [15,16,17,18]—even when already undetectable in respiratory samples or after the resolution of clinical disease [17,19]. Viral quantification in these specimens shows that genome copies are usually high, reaching up to 10^8^ copies per gram of feces, with peak values at the third and fourth weeks of illness [20,21,22]. The duration of detectable RNA in these samples varies among studies, but prolonged RNA detection has already been reported for up to 33 days after the initial symptoms [17,18,23,24]. Nevertheless, it is crucial to elucidate that the detection of viral nucleic acid alone does not effectively denote the presence of infectious SARS-CoV-2. To date, few studies explored and reported cultivable SARS-CoV-2 in these specimens [16,18,25,26], in order to virtually evidence the presence of infectious viral particles in RNA-positive fecal samples. On the other hand, other groups were not successful in viral isolation, despite the high viral RNA concentration in those clinical samples [21]. Altogether, these outcomes suggest a putative fecal–oral role in SARS-CoV-2 transmission, which is still an issue under debate [18,27].

SARS-CoV-2 is shed in human excreta [28] and further disposed in the sewerage or in the environment, in the case of poor basic sanitation settings [29]. Wastewater-based epidemiology (WBE) has been successfully used for detection and monitoring of viral pathogens [30,31], including the WHO acute flaccid paralysis program [32,33,34]. Thus, this approach has also been applied in the context of COVID-19 [34]. This review summarizes the global experience on SARS-CoV-2 wastewater-based epidemiology (WBE) and viral detection in polluted surface water as a valuable tool for COVID-19 surveillance and discusses the advantages and challenges from this strategy as a complementary surveillance tool.

## 2. Global Experience in SARS-CoV-2 WBE Surveillance

The presence of SARS-CoV-2 RNA in wastewater has been reported by many countries, adding information on viral circulation along the distinct pandemic phases, including pre- and post-lockdown assessments. Mostly, detections in wastewater were correlated with local COVID-19 incidence, preceding from 1 to 3 weeks the increase of new clinical cases in the population. These studies also revealed a high rate of RNA detection in different matrices, comprising of raw sewage, inlet and outlet wastewater, sludge and polluted rivers. Some countries adopted the environmental surveillance as part of the routine COVID-19 surveillance—such as Australia, New Zealand, Netherlands [34,35] and parts of Brazil [36,37,38]. This perspective is in course in other countries, such as South Africa [39] and Germany [35].

### 2.1. WBE in the Americas

In Brazil, regular WBE was settled in southeastern (Rio de Janeiro, Brazil; Minas Gerais, Brazil) and southern states (Rio Grande do Sul, Brazil). Thus, the present discussion is mostly based on data from official surveillance sources.

In April 2020, SARS-CoV-2 RNA was detected in 41.7% of wastewater-tested samples from Niteroi city, RJ, reflecting the number of reported COVID-19 cases in that period. In addition, the authors also pointed to the expansion of the outbreak to other areas of the city [40]. Along viral monitoring in wastewater, an increase in detections was observed, reaching 100.0% in late May. Since August, however, a decreasing trend could be observed, in line with the incidence curve in the local population [37]. In Belo Horizonte and Contagem cities, MG, an ongoing investigation assessed SARS-CoV-2 in wastewater samples collected in 24 catchment points from two basins, which attend about 2.2 million inhabitants. The initial results, dated from April 2020, revealed the presence of SARS-CoV-2 RNA in 29.0% and 64.0% of samples from each basin. In June, viral RNA detection extended to 100.0% of the analyzed samples. In accordance with the same trend observed in Niteroi, a significant decline in the number of notified cases has been registered since August [38]. In RS, a regular SARS-CoV-2 surveillance has been conducted in wastewater treatment plants (WWTPs) and water bodies from Porto Alegre city and the metropolitan area, which concentrate about 4.3 million inhabitants. From May to June, viral detection augmented from 12.5% to 83.3%, with a continuous increasing trend until August (100.0%). In order to investigate the viral viability in these samples, two wastewater samples with detectable RNA were further submitted to viral isolation but no cytopathic effect was observed, in line with the non-detectable real time RT-PCR results in infected cell culture supernatants [36]. Of note, in Florianopolis city (Santa Catarina Southern state), an analysis conducted in raw sewage samples, from October 2019 to March 2020, showed the viral RNA presence in 66.6% of samples. According to the authors, SARS-CoV-2 RNA loads raised after late February 2020, which was coincident with the first local COVID-19 official case [41]. Altogether, these figures are consistent with the dynamics of the Brazilian epidemiological scenario. In the southeastern states, the epidemic peak, plateau phase and decreasing trend in the number of new COVID-19 cases and deaths preceded those in the southern states, which present an increasing trend in the epidemic curve since mid-May [42,43].

In the USA, WBE studies were carried out in Massachusetts [44,45], New York [46], Connecticut [47], Montana [48], Virginia [49] and Louisiana [50] states, including wastewater and sludge samples. Viral RNA detection ranged between 13.0 and 100.0% among investigations and was in accordance with the local epidemiological data. Importantly, except for Louisiana, detections in wastewater preceded the identification of new COVID-19 cases in the community. In addition, the magnitude of viral concentration in sludge samples was two to three times higher than those found in raw wastewater, underlining the adequacy of primary sludge samples for SARS-CoV-2 RNA monitoring [47].

In Chile, an investigation was conducted with raw sewage samples from WWTPs from La Farfana and El Trebal, which process about 85.0% of Santiago’s wastewater. SARS-CoV-2 RNA was not detected in March and April collections. However, the detection and quantification of viral RNA progressively increased afterwards, corroborating the COVID-19 incidence in those catchment areas [51].

In Canada, a remarkable study was conducted between April and June 2020, in a decreasing COVID-19 incidence scenario (May to June 2020). The group explored the trends of SARS-CoV-2 quantification in wastewater influent solids (post-grit solids; PGS) and primary clarified sludge (PCS) from two municipal WRRFs (water resource recovery facilities) from Ottawa and Gatineau. In addition, a comparative study between RT-qPCR and RT-droplet digital PCR (R-ddPCR) was carried out. Compared to RT-qPCR, a signal inhibition in RT-ddPCR was found in the PCS samples, which was not observed in PGS, which showed similar RNA concentration in both assays. Viral detection in RT-qPCR was 92.7% and 90.6% among PCS and 79.2 and 82.3%, among PGS samples for N1 and N2 targets, respectively. Thus, PCS can be an adequate sample for viral quantification, even in declining or low COVID-19 incidence contexts. The findings also evidenced that pepper mild mottle virus (PMMV) RNA can be a reliable normalization biomarker, to control noise associated with variances in daily operations, sampling, storage, processing and sample analysis. At last, after normalization of the SARS-CoV-2 signal, a significant correlation was observed between viral RNA gene copies/L and epidemiological data, considering daily new cases, active COVID-19 cases in the last fourteen days, and the percent of daily-testing positives for COVID-19 [52].

### 2.2. WBE in Europe

European countries assembled a key experience and a remarkable collection of SARS-CoV-2 reports. Europe’s first information on SARS-CoV-2 WBE was released by a Dutch group that studied sewage samples from six cities in addition to the Amsterdam airport, from February to March 2020. The investigators originally described the early viral detection in wastewater and that viral load values increased proportionally to disease prevalence [53,54].

In Spain, wastewater surveys were conducted from February to May, 2020 including different localities, as Valencia [55], Murcia [56], Barcelona [57] and Santiago de Compostela [58]. In these studies, SARS-CoV RNA detections ranged from 40.0 to 83.0%, since the earliest stages of COVID-19 epidemics [55]. Detections also preceded the incidence of new COVID-19 cases in the population [55,56,57] and, in conformity with the aforementioned American studies, viral RNA in wastewater matched the estimated cumulative number of clinical cases [55,57].

In France, an investigation monitored the presence of SARS-CoV-2 in wastewater from Paris, from March to April 2020. A high frequency of SARS-CoV-2 RNA detection in raw (100.0%) and treated wastewater (75.0%) was reported [59]. Noteworthy information was further released, describing that lockdown impacted on viral dynamics. After an exponential increasing phase, viral loads reached a peak in early April, followed by a marked decrease in its values. The last was concomitant with a reduction in new COVID-19 cases, attributed to the French lockdown [60]. Another survey, conducted in Montpellier, further explored the re-emergence of SARS-CoV-2 infections by evaluating samples collected from few days before (early May) to 70 days post-lockdown (July). The investigators showed that SARS-CoV-2 viral RNA quantification presented a relevant increase from mid-June on, which preceded the rising number of new COVID-19 patients in some weeks. Despite that, the authors did not find a direct correlation between SARS-CoV-2 RNA levels in wastewater and the number of new COVID-19 cases [61].

In Milan and Rome, analysis of raw sewage samples from the February to April 2020 presented detectable RNA in 50.0% of samples. On late February, epidemics were still limited in Italy and RNA detection in Milan sewage was noticed a few days after the announcement of the first autochthonous case. When SARS-CoV-2 was detected in wastewater samples from Rome, however, epidemics were already established in the country. Hence, the detection of RNA in all wastewater samples was consistent with the epidemiological scenario [62]. La Rosa and colleagues [63] also investigated wastewater samples from Milan, Turin and Bologna, collected between September 2018 and February 2020. The authors evidenced that SARS-CoV-2 was already circulating in Northern Italy at the end of 2019, weeks before the first documented autochthonous case. Eight samples with detectable RNA were collected before the first documented Italian case and the earliest dates address to 18 December 2019 in Milan and Turin and to 29 January 2020 in Bologna, in accordance with Italian molecular epidemiological studies [64].

In Germany, 100.0% of detectable viral RNA was found in nine WWTPs simultaneously investigated in April 2020. Results were further confirmed by Sanger sequencing. In influents, viral loads were one log unit higher in the solid phase, when compared to the aqueous phase (25.0 vs. 1.8 genome copies/mL). As other reports, viral loads in wastewater were proportional to cumulative and acute number of COVID-19 cases in the catchment areas. Samples with detectable RNA were further submitted to viral isolation. No cytopathic effect was observed, reinforcing the low putative risk of infection associated to these matrices, despite their RNA detectable status [65].

In Turkey, it was evidenced a 100.0% SARS-CoV RNA detection in primary sludge (PS) and waste activated sludge (WAS) samples collected in May, 2020 [66]. In line with previous reports [58,65], high SARS-CoV-2 loads were found in these samples, substantiating their adequacy as a matrix for WBE assessments.

### 2.3. WBE in Asia

In Japan, two WBE studies were carried out between March and May 2020, comprising of influent samples from Ishikawa and Toyama and secondary wastewater samples from Yamanashi prefecture. Viral RNA detection varied between 20.0% and 57.0% among influents [67], whereas these figures were 20.0% in secondary-treated wastewater [68]. These findings were in accordance with the regional pattern of local reported cases and showed a suitable WBE sensitivity for viral RNA detection, even in low COVID-19 prevalence scenarios, like Yamanashi.

In China, wastewater assessments revealed a SARS-CoV-2 RNA detections varied between 39.3% and 63.6% [69,70,71]. In one study comprising of outdoor environment samples of three hospitals, viral RNA was found in wastewater and its surrounding soil. According to the authors, it is likely that SARS-CoV-2 arose from viral RNA-containing medical wastewater via aerosolization in the uplifting process, and the aerosols were eventually deposited on the soil. These findings point toward appropriate sealing of wastewater treatment units and complete sanitation to avert putative risks [69]. Noteworthy, viable virus was not recovered from wastewater samples [70].

WBE studies conducted in India showed a high frequency of RNA detection in influent and effluent wastewater, as among hospital sewage samples collected from May to June 2020. In untreated wastewater samples, SARS-CoV-2 RNA presence varied from 75.0 to 100.0%, preceding a significant increase in the number of reported cases in about 10–14 days. However, during late sampling, this time interval decreased, probably due to a progressive reduction in lockdown [72]. According to another study, a 10-fold increase in viral loads was observed between 8 May, 2020 and 27 May, 2020, which corresponded to a two-fold increase in the number of COVID-19 cases [73]. These results also corroborate a correlation between the RNA detection in environmental samples and the trends in the number of new COVID-19 cases.

In Bangladesh, a study was conducted from 10 July to 29 August 2020 in wastewater samples (sewage waste tank, passage drain and toilets), collected in a location near the largest center for COVID-19 patients in the Noakhali district. Viral RNA was detected in 75.0% of samples.

### 2.4. WBE in Oceania and Middle-East

In Australia, viral RNA was detected in 22.2% of untreated wastewater from Queensland, collected in March and April. RNA quantification was used to estimate the number of infected individuals through a Monte Carlo simulation model. The median number of infections ranged from 1090 (first collection) to 171 (second collection) with a median prevalence of 0.096% [74]. In Israel, a preliminary testing of hospital influents and raw sewage revealed the presence of SARS-CoV-2 RNA in 38.4% of samples. Outcomes from Tel Aviv and Jerusalem showed a correspondence between sewage detection and the number of reported cases [75].

## 3. SARS-CoV-2 RNA Detection in Surface Water

Mostly, SARS-CoV-2 detection in wastewater had been assessed in high-resource settings. In lower-resource settings, as sub-Saharan Africa, Indonesia and India, a significant part of the population is not connected to sewers and uses pit toilets, septic tanks or still practices open defecation [29,76,77,78,79]. Waterways are also used as open sewers and the discharge of untreated wastewater into the environment is an usual practice [77,78]. In these contexts, testing of surface water contaminated by sewage could be used as an alternative for viral monitoring [29,35,80].

The presence of SARS-CoV-2 RNA in polluted surface water has been explored by few studies, so far. One of these investigations was performed in June 2020, during the local epidemiological peak of COVID-19 in Quito, Ecuador. Viral RNA was detected in three points from an urban river, which receives the direct discharge of sewage from almost 3 million inhabitants. The frequency of RNA detection and quantification of the viral load clearly paralleled the number of reported cases in each collection area [81]. Corroborating these findings, viral RNA was also encountered in contaminated pretargeted rivers from Rio Grande do Sul, Brazil, due to the high levels of *Escherichia coli*. Among the 14 sampling sites, viral RNA was detected in 44.4% of samples, also resembling the local epidemiological curve of COVID-19 between mid-May and the beginning of September, 2020 [36]. Similar figures were described in Italy. As WWTPs from these locations discharge treated wastewater into Lambro (Milan and Monza, Lombardia, Italy) and Lambro Meridionale (Milan, Lombardia, Italy) rivers, surface water samples were investigated. Viral RNA was detected in both rivers, probably due to non-treated sewage discharges or combined sewage overflows. Of note, viable viruses were not recovered from the Italian assessed samples, suggesting that WWTPs nor rivers compose of significant sources of infective SARS-CoV-2 [82]. In a full contrast to the aforementioned reports, viral RNA was not found in river samples from Yamanashi, Japan [68].

Altogether, these findings suggest that polluted rivers could be an alternative source for SARS-CoV-2 detection and monitoring—a feasible alternative for environmental surveillance, especially in poor sanitation settings and countries with inland unequal sewerage coverage.

Little has been documented on viral viability in environmental samples. This is pivotal information to understand putative associated risks of infection [27,83,84,85,86], since a third of the global population—especially in Central and Southern Asia, Oceania and Sub-Saharan Africa—still lives under critical sanitary conditions, with limited/unimproved sanitation [29,83,87]. SARS-CoV-2 viability and persistence in waste and polluted surface water is still a concern [27,78,84,88,89,90], which demand further research and cumulative data.

The SARS-CoV-2 prevalence in wastewater and polluted rivers, according to the country is summarized in Table 1. Detailed and summarized information for each assessed study—type of samples, sampling method, viral concentration, real time RT-PCR targets, Ct values (cycle threshold), viral concentration and other remarks—can be found as supplementary material (Table S1).

## 4. WBE as a Surveillance Tool: Advantages and Challenges

Components of the public health response include different strategies, such as sentinel surveillance, monitoring of morbidity and mortality rates and clinical-based surveillance [95,96]. In these approaches, the identification and confirmation of suspected cases are based on clinical, epidemiological or laboratory outcomes. In these models, asymptomatic and presymptomatic subjects are hardly identified, especially under a restrict testing context—favoring subnotification and disease burden underestimation [97]. Consequently, WBE can be a remarkable alternative as a complementary surveillance tool, since SARS-CoV-2 detection and monitoring can be performed at the population level [34,35,98]. Evidence of SARS-CoV-2 circulation and/or re-emergence in the community will be critical to reinforce preparedness, to guide public health measures and to limit viral transmission. Moreover, this approach permits the identification of hotspots for the further classical surveillance interventions [35,49,96,99]. In a pandemic scenario, as resources can be severely limited, the use of such a strategy could be pivotal. Thus, WBE presents as a helpful approach to monitor SARS-CoV-2 dynamics and to guide decision-making on lockdown/social distancing and resource targeting [34,35,60,62,100].

Nevertheless, for a successful integration of WBE into a regular SARS-CoV-2 surveillance framework, some issues need to be settled, such as the rate of real time RT-PCR false-positives and negatives in these samples, the effective relationship between viral titers and COVID-19 incidence, and the viral persistence in wastewater and other matrices [34,35,101,102,103]. In addition, the putative impact of extreme weather events—as floods and storms—on wastewater flow variations and viral detection rates remains to be determined. These events are common in tropical areas, such as South America and Africa [83,104,105] and in parts of Asia and Oceania [106].

Other relevant challenges consist on the current knowledge gaps in key aspects of viral disease, like the dynamics of viral shedding in the feces along the infection course, which can directly influence viral loads in wastewater.

**Figure 1:**
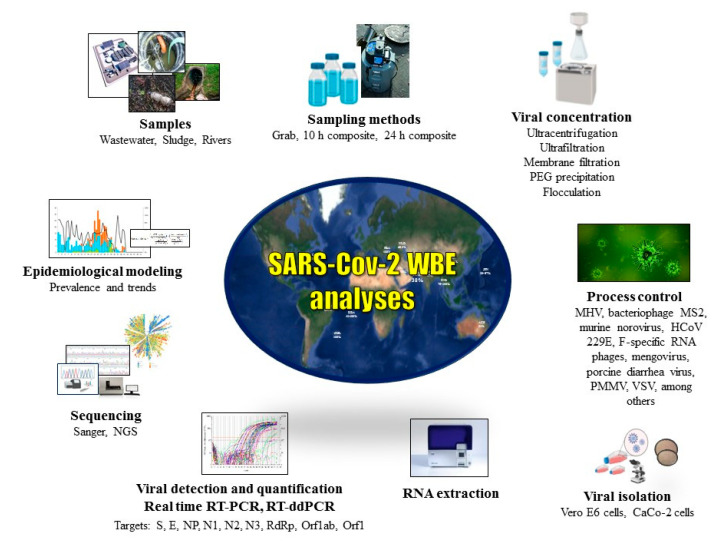
A summary of methods used in the wastewater-based epidemiology (WBE) analyses. PEG, PEG, polyethylene glycol; MHV, murine hepatitis virus; HCoV, human coronavirus, PPMV, pepper mild mottle virus, VSV, vesicular stomatitis virus.

Under the analytical perspective, as presented in the Table S1 and briefly presented in Figure 1, the current SARS-CoV-2 WBE studies used distinct sampling methods, viral concentration techniques, real time RT-PCR targets, process controls and criteria for incidence calculation and interpretation of results. Hence, protocol validation and harmonization of the entire process—from sampling to viral detection—as systematic internal and external quality control are essential tasks to guarantee accuracy, robustness and comparability of results along time and between localities [34,35,90,101,107,108,109,110,111,112]. Recently, Medema and colleagues [34] published a comprehensive and remarkable review, where these issues are debated. Relevant discussions on WBE methodological topics are also presented elsewhere [90,102,107,109,113,114].

## 5. Conclusions

At last, the implementation of a nation-wide WBE program in countries with a dissimilar sanitary coverage is not a trivial issue. The use of distinct sanitation systems, such as centralized sewer systems and on-site sanitation systems—pit latrines, bucket latrines and septic tanks—impose a challenge for WBE implementation in low and middle income countries. Viral RNA detection in dysfunctional sewer systems needs be further explored [29,80,83,84,88].

To the best of our knowledge, an important gap on WBE information in African countries remains. The Water Research Commission from South Africa launched a WBE program to monitor SARS-CoV-2 in the community, divided into three main phases [39]. However, to date, the preliminary findings were not available in the literature. Street [83] argued that the scarcity of accredited laboratories in sub-Saharan countries could contribute to this gap. In addition, the author also emphasized the need of simpler methods for wastewater testing, especially for low-income settings.

Thus, although increasing research data is very motivating and promising, these cavities should be filled for a sustainable surveillance application, as already performed for poliovirus [32,111]. An ideal scenario would be the use of the built-up capacity for environmental surveillance, under the WHO coordination and leadership, as part of the COVID-19 public health response [115].

WBE is an effective approach for early identification and monitoring of SARS-CoV-2 temporal and geographical trends and requires a collaborative effort to be effective [110]. Thus, it is a valuable complementary surveillance tool to subside public health response along pandemics, to tailor containment and mitigation measures and to determine target populations for testing. Validation and harmonization of sampling and analytical methods are critical to improve comparability and to assure accurate and reproducible outcomes. Associated-costs, built up capacity, transparency and accountability appear as catalysts for WBE accomplishment. In addition, available resources should be considered within this perspective for an affordable, sustainable and successful WBE implementation in high-, middle- and low-income countries. Available data suggest that rivers polluted by waste disposal and sewage discharge could be alternatively used as a source for environmental surveillance.

Future research is required to fill knowledge caveats, such as the kinetics of viral shedding in feces and other excreta, SARS-CoV-2 infectivity in stool, surface water, wastewater and other matrices, viral persistence and infectivity in the distinct environmental contexts and the effective feasibility of fecal–oral transmission, especially in middle- and low-income countries. Gwenzi, W. presented a comprehensive discussion in this matter [84]. To date, no infectious SARS-CoV2 virus has been recovered from drinking water supplies, untreated or treated sewage or surface water [36,65,70,82,116]. Cumulative evidence is critical to understand the putative role of these elements as potential sources of viral transmission, particularly in poor sanitation settings [76,78,85,86,117].

Increasing evidence support the value of genomic surveillance as a powerful tool to identify emerging pathogens of public health relevance and to track patterns of viral dispersion [118]. Hence, both assessments should be integrated into a single surveillance framework.

Finally, in full agreement with Adelodun and colleagues [78], investments in environmental surveillance, including the systematic empowering of laboratory networks, should be considered as a priority for middle- and low-income countries.

## Figures and Tables

**Table 1:** Global studies on SARS-CoV-2 prevalence in wastewater and rivers, according to the country, January to August 2020.

Country	Localities	Period	Type of Water Sample	Main Outcomes and Remarks	References
Brazil	Rio de Janeiro, Minas Gerais, Rio Grande do Sul, Santa Catarina	Oct. 2019–Aug, 2020	Raw sewage, treated effluents, hospital wastewater and sewers network. Rivers	Viral RNA detection was 12.5–100.0% and 44.4% in wastewater and rivers, respectively (3 ongoing studies). Viral isolation was performed in two samples, with non-cultivable virus or detectable results. Wastewater detection was in line with clinical and epidemiological data	[36,38,40,41]
Chile	Santiago	Mar–Jun, 2020	Influent and effluent wastewater (WWTPs ^1^)	Viral RNA detection along time: 100.0%. SARS-CoV-2 RNA was not detected during Mar and Apr 2020, but SARS-CoV-2 viral load progressively increased from May to June	[51]
Ecuador	Quito	Jun, 2020	Rivers	Viral RNA detection: 100.0% Wastewater detection was in line with clinical and epidemiological data	[81]
Canada	Ottawa and Gatineau	Apr–Jun, 2020	Influent post grit solids (PGS) Primary clarified sludge (PCS)	Viral RNA detection was 92.7% and 90.6% among PCS samples and 79.2 and 82.3%, among PGS samples for N1 and N2 targets, respectively. RT-qPCR shows higher frequency of detection of N1 and N2 genes in PCS (92.7, 90.6%) as compared to PGS samples (79.2, 82.3%) RT-qPCR shows superior quantification of SARS-CoV-2 PCS compared to RT-ddPCR After normalization, significant correlations were observed between gene copies/L and epidemiological data	[52]
USA	Connecticut, Massachusetts, Montana, New York, Virginia, Louisiana	Jan–Jul, 2020	Raw sewage, influents, daily primary sludge samples from WWTP	Viral RNA detection: 13.0%–100.0% Viral titers in wastewater increased exponentially from mid-March to mid-Apr, followed by a peak on the beginning of April and a subsequent decline. Wastewater detection was in line with clinical and epidemiological data Early detection: RNA detection in wastewater preceded COVID-19 cases in 1–10 days	[44,45,47,48,49,50]
China	Wuhan	Feb–Apr, 2020	Raw sewage, influent and effluent wastewater from septic tanks	Viral RNA detection: 39.3%–60% in sewage samples; 0%–63.6% in effluents. No SARS-CoV-2 was detected in effluents after disinfection Viral isolation was performed, with non-detectable results.	[69,70,71]
India	Jaipur	May–Jun, 2020	Influent and effluent wastewater samples and hospitals	Viral RNA detection: 75.0%–100.0% in influent samples; RNA was undetectable in untreated wastewater Early detection: RNA detection in wastewater preceded the increase in reported cases in 10–14 days	[72,73]
Japan	Ishikawa, Toyama and Yamanashi	Mar–May, 2020	Influent and secondary-treated wastewater Rivers	Viral RNA detection: 20.0%–57.0% in influents; 20.0% in secondary-treated wastewater. SARS-CoV-2 RNA was not detected in rivers. Authors reported wastewater detection even in scenarios of low COVID-19 incidence.	[67,68]
Pakistan	Islamabad	Mar–Apr, 2020	Sewage	Viral RNA detection: 27.0% (21/78)	[91]
Bangladesh	Noakhali	Jul–Aug, 2020	Wastewater (sewage waste tank, passage drain, and toilets)	Viral RNA detection: 75.0% (12/16)	[92]
Australia	Queensland	Mar–Apr, 2020	Untreated wastewater	Viral RNA detection: 22.2% (2/9)	[74]
France	Paris, Montpellier	Mar–Jul, 2020	Raw sewage, influent, effluent wastewater (WWTP)	Viral RNA detection: 100.0% in raw wastewater and 75.0% in treated wastewater samples Wastewater detection was in line with clinical and epidemiological data After a 2-log exponential increase, viral load reached a peak, followed by a marked decrease. RNA detection preceded the exponential growth of the epidemic. Post-lockdown, a fifty-fold increase in SARS-CoV-2 RNA genome copies was observed from mid-June samples, about a month after the end of lockdown. Early detection: RNA detection in wastewater preceded the increase of new COVID-19 cases in 2–3 weeks and the exponential growth of the epidemics	[59,60,61]
Italy	Milan, Monza, Rome, Turin/Piedmont and Bologna/Emilia Romagna	Feb–Apr, 2020	Raw and treated wastewater Rivers	Viral RNA detection: 50.0%–100.0% in wastewater and all polluted river samples were positive. Treated wastewater showed undetectable viral RNA. Viral isolation was performed with non-detectable results. Early detection: RNA detection in wastewater preceded the first reported cases. SARS-CoV-2 was in circulation in Northern Italy at the end of 2019. The earliest dates back to December 18, 2019 in Milan and Turin and January 29, 2020 in Bologna (retrospective study)	[62,63,82]
Netherlands	Amsterdam, Hague, Utrecht, Apeldoorn, Tilburg and Schiphol airport	Feb–Mar, 2020	Wastewater samples	Viral RNA detection: 68.9%. Early detection: RNA detection in wastewater preceded the first clinical case in 4–6 days	[54,93]
Spain	Murcia, Totana, Lorca, Cartagena, Cieza, Molina de Segura, Valencia, Barcelona, Santiago de Compostela	Feb–May, 2020	Raw sewage, influents, secondary and tertiary treated effluent water, sludge. Frozen archival samples from 2018 (Jan-Mar), 2019 (Jan, Mar, Sep-Dec) and 2020 (Jan-Mar)	Viral RNA detection: 40.0–83.0% in wastewater, 40.0% in sludge, 11.0% in secondary treated water samples. No tertiary effluent samples were positive. Early detection: RNA detection in wastewater preceded clinical cases in 12–16 days	[55,56,57,58]
Germany	North Westphalia	8 April, 2020	Wastewater Sludge	Viral RNA detection: 100.0% but some positives were not confirmed by Sanger sequencing. In influents, SARS-CoV-2 RNA gc/mL was one log unit higher in the solid phase (25 vs.1.8 gc/mL) The total load of gene equivalents in wastewater correlated with the cumulative and the acute number of COVID-19 cases reported in the respective catchment areas Viral isolation: negative	[65]
Czech Republic	Different localities, not reported	Apr–Jun, 2020	Raw wastewater	Viral RNA detection: 11.6%	[94]
Turkey	Istanbul	7 May, 2020	Wastewater Primary and activated sludge	Viral RNA detection: 100.0%. Viral concentration was similar in primary and activated sludge samples	[66]
Israel	Tel Aviv	NR	Raw sewage, hospital effluents	Viral RNA detection: 38.4%	[75]

^1^ WWTPs, wastewater treatment plants; NR, not reported.

## Supplementary Materials

The following are available online at https://www.mdpi.com/1660-4601/17/24/9251/s1, Table S1. Global studies on SARS-CoV-2 prevalence in wastewater and rivers, according to country, January to August 2020.
