# Supplementary material for: Wastewater-Based Epidemiology (WBE) and Viral Detection in Polluted Surface Water: A Valuable Tool for COVID-19 Surveillance—A Brief Review
Source: Int J Environ Res Public Health. 2020 Dec 10;17(24):9251. doi: 10.3390/ijerph17249251 (PMC7764684; doi:10.3390/ijerph17249251)
Supplement: Supplementary file 1 [file ijerph-17-09251-s001.pdf]

Supplementary Table 1. Global studies on SARS-CoV-2 prevalence in wastewater and rivers, according to country, January to August 2020

Country (state/city)	Study design					Main outcomes and remarks		Reference
	Period	Type of water sample	Sampling method (N)	Virus concentration method	Real time RT-PCR[1] viral targets	Ct[2] value range and/or viral load	Main outcomes	
Brazil, Florianopolis	Oct, 2019 - Mar, 2020	• Raw sewage	Grab (N=6)	PEG[3] precipitation and filtration	N1, S, RdRp	5.49 ± 0.02 to 6.68 ± 0.02 gc/L[4]	• RNA was undetectable in October and early November, 2019, but all samples were positive since 27 Nov, 2019<br>• Early detection: RNA detections preceded the first case in 91 days	[41]
Brazil, Minas Gerais	13 Apr, 2020 (ongoing)	• Raw sewage and treated effluents (24 catchment points from two basins, including 15 sewage sub-basins, 4 WWTPs[5] and 3 hospitals)	Grab and composite (variable)	Membrane filtration and direct extraction	NR[6]	NR	• Viral RNA detection: 29.0-64.0% in initial catchments to 100.0% in the last weeks	[38]
Brazil, Rio de Janeiro	5 Apr, 2020	• WWTP, Sewage treatment plants (STPs), hospital wastewater and sewers network	10h-composite (N=12)	UC[7]	N2	Ct 36.3-39.8	• Viral RNA detection: 41.7% (5/12)	[40]
Brazil, Rio Grande do Sul	11 May (ongoing)	• WWTP, hospital wastewater, Wastewater Pumping Station<br>• Rivers	Grab and 24h or 6h composite (N=42)	UC	E, N1, N2	Ct 23.0-38.0 / 6,00E+04 to 6,5E+05 gc/L	• Viral RNA detection: 12.5% to 100.0% in wastewater and 44.0% in polluted rivers.<br>• Viral isolation: negative	[36]
Chile, Santiago	Mar - Jun, 2020	• Influent and effluent wastewater from 2 WWTPs	24h-composite (N=4)	UC	ORF1ab,S, N	Ct 28.1 to 37.7/ up to 5000 genome equivalent/ml	• Viral RNA detection along time: 100.0%.<br>• SARS-CoV-2 RNA was not detected during March and April 2020, but SARS-CoV-2 viral load progressively increased from May to June	[51]

Ecuador, Quito	5 Jun, 2020	• Rivers, direct discharge of sewage water	Grab (N=3)	Milk flocculation	N1, N2	2.1×10^5 to 8.5×10^5 gc/L	• Viral RNA detection: 100.0% (3/3)	[81]
USA, Connecticut	19 Mar - 1 May, 2020	• Daily primary sludge samples from WWTP	Grab (N=44)	Direct method	N1, N2	1.7×10^3 gc/mL-1 to 4.6 x 10^5 gc/mL-1	• Viral RNA detection: 100.0%<br>• Early detection: RNA detection preceded hospital admissions in 1-4 days	[47]
USA, Massachusetts	18 - 25 Mar, 2020	• Raw sewage influent	24hr - composite (N=14)	PEG precipitation with pre-filtration (0.2 um)	S, N1, N2, N3	Ct 33.8-38.4/up to ~250 gc/ml	• Viral RNA detection: 100.0% (4/4)<br>• Early detection: RNA detection preceded the first US known cases	[44]
USA, Massachusetts	Jan - May, 2020	• Raw sewage influent (11 composite; 23 grab samples x 4 days)	Grab and 24hr-composite (n=136)	PEG precipitation with pre-filtration (0.2 um) and UC	S, N1, N2	≤ 15 to 2000 gc/ml	• Viral titers in wastewater increased exponentially from mid-March to mid-April, declining afterwards<br>• Early detection: RNA detection preceded the first cases in 4-10 days	[45]
USA, Montana	Mar -Jun, 2020	• Untreated wastewater	Grab and 24h - composite (N=52)	Sequential filtration (20 um, 5 um, 0.45 um), ultrafiltration	N1, N2	10^4 -10^6 gc/L	• Viral RNA detection: 50.0% (6/12)<br>• Early detection: RNA detection preceded the first cases in 1-2 days	[48]
USA, New York	6 May and 13 May, 2020	• Wastewater (11 points WWTP, pump stations, interceptor lines), 2 time points	Grab and 24h - composite (N=22)	UC	IP2, IP4	42.7 to 112.3 gc/ml	• Viral RNA detection: 81.1% (18/22)	[46]
USA, Virginia	Mar-Jul, 2020	• Raw wastewater influent samples from 9 WWTPs (weekly collected)	24h – composite (N=198)	InnovaPrep Concentrating Pipette Select and electronegative filtration	N1, N2, N3 (digital RT-PCR)	10^1 to 10^4 gc/100 mL^{-1}	• Viral RNA detection: 49.5% (98/198).<br>• A sharp increase in detections was perceived in mid-March for 3 three weeks, followed by a peak on the beginning of April and a further decline. Since the end of April, a new increase in the detection frequency has been observed.	[49]
USA, Louisiana	Jan-Apr, 2020	• Wastewater (untreated, secondary treated and effluents)	Grab, 24h composite (N=15)	Ultrafiltration and adsorption–elution (electronegative membrane)	N1,N2	3.1×10^3 - 7.5×10^3gc/L	• Viral RNA detection: 13.0% (2/15)	[50]

Canada, Ottawa and Gatineau	Apr-Jun, 2020	• Influent post grit solids (PGS)<br>• Primary clarified sludge (PCS)	Grab, 24-hour composite	Serial filtration and PEG precipitation	N1,N2 E-Sarbeco (qRT-PCR, one-step RT-ddPCR)	1.7 x 103 to 7.8 x 104 copies//L (Ottawa) and 6.6 x 104 to 3.8 x 105 454 copies/L (Gatineau)	• Viral RNA detection: 92.7% and 90.6% among PCS samples and 79.2 and 82.3%, among PGS samples for N1 and N2 targets, respectively.<br>• RT-qPCR shows higher frequency of detection of N1 and N2 genes in PCS (92.7, 90.6%) as compared to PGS samples (79.2, 82.3%)<br>• RT-qPCR shows superior quantification of SARS-CoV-2 PCS compared to RT-ddPCR<br>• After normalization, significant correlations were observed between gene copies/L and epidemiological data	[52]
China	19 - 24 Feb, 2020	• Sewage (isolation ward sewage; inlets of hospital sewage disinfection pool, preprocessing disinfection pool; outlet)	Grab (N=5)	NR	NP	Ct 29.3-33.5	• Viral RNA detection: 60.0% (3/5) in sewage samples (inlet of preprocessing disinfection pool). The sample from the outlet of the last disinfection pool was negative.<br>• Viral isolation: negative	[70]
China	Feb - Mar, 2020	• Influent and effluent wastewater from septic tanks	Grab (N=15)	PEG precipitation	ORF1, N	30.1-35.7	• Viral RNA detection: 63.6% (7/9) in effluents. Undetectable RNA in influents (0/4)	[71]
China, Wuhan	Mar - Apr, 2020	• Wastewater (samples from treatment tanks, adjusting tank, raw medical wastewater, sedimentation tank)	Grab (N=28)	PEG precipitation	ORF1, N	255 to 18,7 gc/L	• Viral RNA detection: 39.3% (11/28). No SARS-CoV-2 was detected in effluents after disinfection	[69]
India, Jaipur	3 May and 14 Jun, 2020	• Influent and effluent wastewater samples and hospitals	Grab, composite (N=13)	PEG precipitation and direct method	S, E, N RdRp, ORF1ab,	Ct 23.7-36.0	• Viral RNA detection: 75.0% (6/8) in influents<br>• Early detection: RNA detection preceded the increase in reported cases in 10-14 days	[72]

India, Jaipur	8 and 27 May, 2020	• Wastewater samples	Grab (N=4)	PEG precipitation and 96 well filter plate	ORF1ab,N, S	Ct 32.6-35.9/ 2.4×10^{8}gc/L	• Viral RNA detection: 100.0% in untreated wastewater. None positives in treated wastewater	[73]
Japan, Ishikawa and Toyama	5 Mar - 23 Apr, 2020	• Influent wastewater samples	Grab (N=27)	PEG precipitation	N2, N3	1.2×10^{4} to 4.4×10^{4} gc/L.	• Viral RNA detection: 25.9% (7/27). In influents, 20.0% - 57.0%.	[67]
Japan, Yamanashi	17 Mar and 7 May, 2020	• Influent and secondary-treated wastewater<br>• River	Grab (N=13)	Electronegative membrane; membrane adsorption-direct RNA extraction	N-Sarbeco, N1, N2, ORF1a, S	Influent: 3.2 to $5.0\text{x}10^{7}$; Rivers: 1.8 to $4.0\text{x } 10^{5}$ gc/L	• Viral RNA detection: 20.0% (1/5) in secondary-treated wastewater. RNA was undetectable in all influent and rivers samples<br>• Detections occurred in a low incidence scenario	[68]
Pakistan, Islamabad	20 Mar - 28 April, 2020	• Sewage	Grab (N=78)	Centrifugation and PEG-dextran precipitation	ORF1ab, ORF-1, N, E	Ct 32.0-38.0	• Viral RNA detection: 27.0% (21/78)	[91]
Bangladesh, Noakhali	10 Jul – 29 Aug, 2020	• Wastewater (sewage waste tank, passage drain, and toilets)	Grab (N=16)	Flitration and PEG precipitation	ORF1ab, N	Ct 20.4-40-7	• Viral RNA detection: 75.0% (12/16)	[92]
Australia, Queensland	20 Mar – 1 Apr, 2020	• Untreated wastewater	Grab and composite (N=9)	Electronegative membrane; ultrafiltration	N-Sarbeco, NIID_2019 -nCOV_N	Ct 37-40/1.9 to 12 gc/100mL	• Viral RNA detection: 22.2% (2/9)	[74]
France, Paris	Mar - Apr, 2020	• Raw sewage influent	Grab (N=27)	UC	E-Sarbeco	5.10^{4} gc/L - 3.10^{6} gc/L	• Viral RNA detection: 100.0% (27/27). After a 2-log exponential increase, viral loads reached a peak, followed by a marked decrease.<br>• Early detection: RNA detection preceded the exponential growth of the epidemic	[60]
France, Paris	5 Mar - 7 Apr, 2020.	• Raw and treated wastewater	Grab (N=31)	UC	E	10^{4} -10^{7} eq/L	• Viral RNA detection: 100.0% in raw wastewater and 75.0% (6/8) in treated wastewater samples.<br>• Early detection: RNA detection preceded the exponential growth of the epidemic	[59]

France, Montpellier	May-Jul, 2020	• Effluent wastewater (WWTP)	24h – composite (N=7)	Membrane filtration	N1, N3	10^2-10^4 gc/ml	• A fifty-fold increase in SARS-CoV-2 RNA genome copies was observed since mid-June samples, about a month after the end of lockdown Early detection: RNA detection preceded the increase of new COVID-19 cases in 2-3 weeks	[61]
Italy, Milan and Monza	14 and 22 Apr, 2020	• Raw and treated wastewater<br>• Rivers	Grab (N=8)	Glass fiber filtration and membrane filtration	N, ORF1ab, E	NR	• Viral RNA detection: 100.0% in raw wastewater and river samples. RNA was undetectable in all treated wastewater showed negative results.<br>• Viral isolation: negative	[82]
Italy, Milan and Rome	3 Feb - 2 Apr, 2020	• Raw sewage	24h - composite (N=12)	PEG-dextran precipitation	ORF1ab, S, RdRP	NR	• Viral RNA detection: 50.0% (6/12)<br>• Positive in Milan was collected a few days after the first case.	[62]
Italy, Milan/Lombardy, Turin/Piedmont and Bologna/Emilia Romagna	9 Oct, 2019 - 28 Feb, 2020	• Influent wastewater samples and retrospective samples from 5 WWTPs	24h - composite (N=40)	WHO modified two-phase (PEG-dextran)	E, RdRp, E-Sarbeco, ORF1ab	Undetectable to 5.6 × 10^4 gc/L, most results 10^2-10^3 gc/L.	• Early detection: RNA detection preceded the first reported cases. SARS-CoV-2 was in circulation in Northern Italy at the end of 2019. In Milan and Turin, the earliest dates back to December 18, 2019 and, in Bologna, to January 29, 2020	[63]
Netherlands, Amsterdam, Hague, Utrecht, Apeldoorn, Tilburg and Schiphol airport	5 Feb - 25 Mar, 2020	• Sewage	24h - composite (N=30)	Ultrafiltration (Centricon)	N1, N2, N3,E	29.9-37.8/ 2.6–30 gc/mL (early) to 790–2200 gc/ml	• Viral RNA detection: 68.9%.<br>• Early detection: RNA detection preceded the first reported case in 6 days	[54]
Netherlands, Amsterdam	Feb - Apr, 2020	• Wastewater	24h - composite (NR)	NR	NR	NR	• Early detection: RNA detection preceded the first cases in 4 days	[93]

Spain Murcia, Totana, Lorca, Cartagena, Cieza, Molina de Segura	12 Mar - 14 Apr, 2020	• Influent, secondary and tertiary treated effluent water	Grab (N=72)	Adsorption-precipitation (aluminum hydroxide)	N1, N2, N3	Ct 34.0-37.8 / Untreated wastewater: 5.1 ± 0.3 log10 gc/L-5.5 ± 0.3 log10 gc/L	• Viral RNA detection: 83.0% (35/42) in influent samples and 11% (2/18) in secondary treated water samples. RNA was undetectable in tertiary effluent samples (0/12).<br>• Early detection: RNA detection preceded the reported cases in 12–16 days	[56]
Spain, Valencia	12 Feb - 14 Apr, 2020	• Wastewater (WWTPs)	Grab (N=24)	Flocculation precipitation	N1, N2	Ct 34.0-37·8 5.22 and 5.99gc/L	• Viral RNA detection: 80.0% (12/15)<br>• Early detection: RNA detection preceded the first declared case in Spain in 1 day	[55]
Spain, Barcelona	13 Apr - 25 May, 2020	• Raw sewage samples from WWTP; frozen archival samples from 2018 (Jan-Mar), 2019 (Jan, Mar, Sep-Dec) and 2020 (Jan-Mar)	Grab and 24h - composite (N=28)	PEG precipitation	RdRp, IP2, IP4, E, E-Charité, N1, N2	2 to 5 log 10 gc/L	• Early detection: RNA detection preceded the declaration of the first case in 41 days	[57]
Spain, Santiago de Compostela	6 Apr - 21 Apr, 2020	• Wastewater and sludge	Grab and 24h - composite (N=50)	Ultrafiltration; PEG precipitation	RdRP, N, E	Ct 33.7-39.8 / Influent: 7.5 to 15 gc/ml. Sludge: 10 to 40 gc/ml	• Viral RNA detection: 40.0% (6/15) in wastewater and 40.0% (14/35) in sludge	[58]
Germany, North Westphalia	8 April, 2020	• Wastewater and sludge	24h - composite	Ultrafiltration	RdRP,M, N, E	Ct 32-37/Influent: 3.0 and 20 gc/ml Effluent: 2.7 to 37 gc/ml	• Viral RNA detection: 100.0% but some positives were not confirmed by Sanger sequencing.<br>• In influents, SARS-CoV-2 RNA gc/ml was one log unit higher in the solid phase (25 vs.1.8 gc/mL)<br>• The total load of gene equivalents in wastewater correlated with the cumulative and the acute number of COVID-19 cases reported in the respective catchment areas<br>• Viral isolation: negative	[65]

Czech Republic	Apr - Jun, 2020	• Raw wastewater (33 WWTPs)	24h - composite (N=112)	Direct flocculation	ORF1ab, E	Ct 34-40	• Viral RNA detection: 11.6%	[94]
Turkey, Istambul	7 May, 2020	• Wastewater, primary and activated sludge	Grab (N=9)	PEG precipitation	RdRp	Ct 33.5 to 35.8 / 1.17×10^4 to 4.02×10^4 gc/ml	• Viral RNA detection: 100.0% (9/9). Viral concentration was similar in PS and WAS samples	[66]
Israel, Tel Aviv	NR	• Raw sewage, hospital effluents	Grab and 24h - composite (N=26)	PEG precipitation or alum precipitation; ultrafiltration	E	Ct 32.8-38.03	• Viral RNA detection: 38.4%	[75]

[1] RT-PCR, reverse-transcription followed by polymerase chain reaction; [2] Ct, cycle threshold; [3] PEG, polyethilene glycol; [4] gc, genome copies; [5] WWTPs, wastewater treatment plants; [6] NR, not reported; [7] UC, ultracentrifugation
